# Use of healthcare services and therapeutic measures associated with new episodes of acute low back pain-related disability among elderly people: a cross-sectional study on the Back Complaints in the Elders - Brazil cohort

**DOI:** 10.1590/1516-3180.2020.0414.R1.0712020

**Published:** 2021-04-05

**Authors:** Juleimar Soares Coelho de Amorim, Vitor Tigre Martins Rocha, Lygia Paccini Lustosa, Leani Souza Máximo Pereira

**Affiliations:** I PhD. Physiotherapist and Associate Professor, Physical Therapy Course, Instituto Federal de Educação Ciência e Tecnologia do Rio de Janeiro (IFRJ), Rio de Janeiro (RJ), Brazil.; II MD. Physiotherapist, Postgraduate Program on Rehabilitation Sciences, Department of Physical Therapy, Universidade Federal de Minas Gerais (UFMG), Belo Horizonte (MG), Brazil.; III PhD. Physiotherapist and Associate Professor, Postgraduate Program on Rehabilitation Sciences, Department of Physical Therapy, Universidade Federal de Minas Gerais (UFMG), Belo Horizonte (MG), Brazil.; IV PhD. Physiotherapist and Associate Professor, Postgraduate Program on Rehabilitation Sciences, Department of Physical Therapy, Universidade Federal de Minas Gerais (UFMG), Belo Horizonte (MG), Brazil.

**Keywords:** Low back pain, International Classification of Functioning, Disability and Health, Disability studies, Health services for the aged, Elders. Disability, Disability-related low back pain, Doctor visits, Crowding-in effects

## Abstract

**BACKGROUND:**

Patients with low back pain frequently undergo a variety of diagnostic and therapeutic interventions, but some of these have uncertain effectiveness. This highlights the importance of the association of healthcare services and therapeutic measures relating to disability.

**OBJECTIVE:**

To analyze the use of healthcare services and therapeutic measures among Brazilian older adults with disability-related low back pain.

**DESIGN AND SETTING:**

Observational cross-sectional study on baseline assessment data from the Back Complaints in the Elders - Brazil (BACE-B) cohort.

**METHODS:**

The main analyses were based on a consecutive sample of 602 older adult participants in BACE-B (60 years of age and over). The main outcome measurement for disability-related low back pain was defined as a score of 14 points or more in the Roland Morris Questionnaire.

**RESULTS:**

Visits to doctors in the previous six weeks (odds ratio, OR = 1.82; 95% confidence interval, CI 1.22-2.71) and use of analgesics in the previous three months (OR = 1.57; 95% CI 1.07-2.31) showed statistically significant associations with disability-related low back pain. The probability of disability-related low back pain had an additive effect to the combination of use of healthcare services and therapeutic measures (OR = 2.57; 95% CI 1.52-4.36). The analyses showed that this association was significant among women, but not among men.

**CONCLUSIONS:**

Occurrence of the combined of consultations and medication use was correlated with higher chance of severe disability among these elderly people with nonspecific low back pain. This suggested that overuse and “crowding-in” effects were present in medical services for elderly people.

## INTRODUCTION

Disabilities caused by chronic-degenerative diseases and their symptoms, such as spinal problems, combined with the aging population, are a major challenge to healthcare systems around the world.^1^^,^^2^ Low back pain is the single biggest cause of years lived with disability worldwide and is the condition that most contributes to overall disability and life years with disability.^3^^,^^4^

In Brazil’s National Household Sampling Survey (Pesquisa Nacional por Amostra de Domicílio), performed in 2003, back pain was identified as the most prevalent chronic disease. Its incidence increased with increasing age, to reach 40% among older adults between 69 and 79 years of age.^5^ The point-prevalence of low back pain was found to be 25.0% among elderly Brazilians in a systematic review, and other studies indicated its potential to affect the functions, activities and social participation of older adults, with concomitant impacts on quality of life and independence.^4^^,^^6^^,^^7^^,^^8^

Longitudinal analyses have shown that after one year, 60.3% of older adults with low back pain continue to present disability. After two years, the disability-related low back pain of only 36% of older adults has halved, which demonstrates the long-term latency of such complaints.^9^^,^^10^ It has consequently been observed that the high prevalence of low back pain, its related disability and the poor long-term prognosis induces older adults to request specific interventions.^11^ Among community-dwelling elderly people, about 58% seek healthcare due to low back pain.^12^ Patients with back pain frequently undergo a variety of diagnostic and therapeutic interventions (imaging tests, injections, pain medications, physical therapy, surgery, braces, etc.), and some of these have uncertain effectiveness.^13^^,^^14^^,^^15^^,^^16^^,^^17^ Elderly people with severe disability have been found to be 3.8 times more likely to be seeking care, and factors like gender, histories of previous low back pain and general health also influence care-seeking behavior.^12^

Recent reports have shown that people with the poorest physical and mental health are significantly more likely to present associated low back pain-related disability.^18^^,^^19^ The causes of use of healthcare services and therapeutic measures are related to context (types of healthcare systems and their organization, for example) and individual factors (age, sex, education and income).^20^^,^^21^ According to the classical model of Andersen and Newman, individual factors include predisposing factors (age and sex),^21^ enabling characteristics (like education level and income) and healthcare needs from the perspectives of professionals and users. Thus, the findings from the existing studies cannot be extrapolated to populations in low and middle-income countries, such as Brazil, due to differences in the setup and planning of their healthcare systems.

Studies on the utilization of healthcare services and therapeutic measures among older adults with disability-related low back pain remain at an initial stage. Therefore, it was sought through the present study to investigate the healthcare services and therapeutic measures that are used in relation to complaints of acute low back pain-related disability among community-dwelling elderly people. We hypothesized that the associations between the characteristics of use of healthcare and therapeutic measures and occurrences of disability would differ between the sexes.

## OBJECTIVE

In this study, data from the Back Complaints in the Elders - Brazil (BACE-B) cohort were used. The aim was to analyze associations between the use of healthcare services and therapeutic measures and occurrences of disability reported by elderly people.

## METHODS

### Study design and ethics

This study forms part of an international consortium of epidemiological studies named Back Complaints in the Elders, which includes researchers in Australia, Brazil and the Netherlands.^22^ The Back Complaints in the Elders - Brazil project is a prospective cohort study with data collected between October 2011 and September 2015. The present analysis was an observational cross-sectional study on baseline assessment data from the Back Complaints in the Elders - Brazil cohort. Ethical approval for this study was obtained from the Ethics Committee of the Federal University of Minas Gerais (Universidade Federal de Minas Gerais, UFMG), under the number ETIC 0100.0.203.000-11, on February 24, 2016.

### Study population

The sample for this study comprised consecutive participants in the baseline survey for the Back Complaints in the Elders - Brazil cohort. They were aged ≥ 60 years, with acute complaints of low back pain, and were residents in the metropolitan region of Belo Horizonte, Minas Gerais, Brazil. Elderly people with low back pain symptoms were identified by healthcare professionals (physicians, physiotherapists and occupational therapists, among others) working in either the public or the private healthcare sector, and were directed to the BACE-B research team.

Only elderly people presenting criteria for a new episode of acute low back pain were included in the Back Complaints in the Elders - Brazil study. Low back pain was defined as complaints of pain, tension or stiffness in the region between the last ribs and the gluteal line, with or without irradiation of pain to the lower limbs.^23^ A new episode of low back pain was defined as a situation in which the individual had not sought treatment for low back pain over a six-month period immediately preceding participation in the study.^22^ Acute symptoms were defined as an occurrence of a low back pain crisis not more than six weeks before the baseline assessment.^24^

Participants with visual, motor, hearing or cognitive impairment that could influence their responses to questionnaires or prevent adequate performance in physical and functional tests were excluded.^25^

### Outcome variable

The outcome measurement of this study was disability-related low back pain, as assessed using the Roland Morris Disability Questionnaire (RMDQ), which consists of 24 items relating to the influence of back pain on daily activities and measures the level of disability associated with low back pain. The questionnaire scores range from 0 to 24, with higher scores indicating a worse level of disability.^26^^,^^27^ Scores over 14 were taken to indicate severe disability.^27^

### Exposure variable

Five indicators of use of healthcare services were considered: doctor visits made over the preceding six weeks, including generalist, specialist and occupational doctors; physiotherapy consultations over the preceding six weeks; diagnostic tests made during the preceding three months, including blood tests, X-rays, computed tomography and magnetic resonance imaging; and use of analgesics and complementary noninvasive therapies (orthoses, braces, acupuncture, yoga, Pilates, overall postural re-education or other types).

### Sociodemographic and back pain variables

Potential confounding variables were selected for this analysis based on the theoretical model of Andersen and Newman,^21^ in which predisposing and enabling factors for the use of healthcare services were considered. Among the predisposing factors, sex and age (continuous variable) were considered. Among the enabling factors, living with a spouse/partner (yes or no), educational level/schooling years (less than four; or four or more) and own income (up to one minimum monthly wage, two to four, or five or more) were considered.

The low back pain intensity in the last week was evaluated by means of a numerical rating scale (NRS), on which the scores could range from 0 (no pain) to 10 (maximum pain).

### Statistical analysis

Descriptive analyses were performed on the outcomes investigated, both for the total population and with stratification according to sex, using proportions and means (with standard deviation). Comparisons between groups were made using Pearson’s chi-square or Fisher’s exact test (for proportions), or using Student’s t test (for means).

Multivariate analysis was done to investigate associations between disability and indicators of use of healthcare services and therapeutic measures and was based on odds ratio (OR) estimates, by means of binary logistic regression. The multivariate models were adjusted for age, sex, living with spouse/partner, education level and income. Binary logistic regression was used to estimate the predicted probability of occurrences of doctor visits over the preceding six weeks and use of analgesic medications over the preceding three months, according to disability.

All the analyses were performed using the procedures for complex samples in the Stata statistical package, version 13.0 (StataCorp LLC, College Station, TX, United States), with a significance level of 5%.

## RESULTS

**Figure 1.**
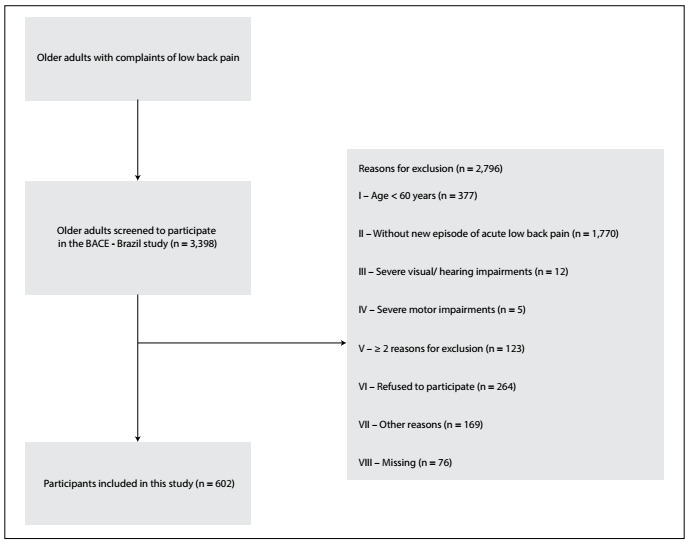
Flowchart of entry of participants into the study.

The sample consisted of 602 elderly people with complaints of acute nonspecific low back pain. Figure 1 illustrates the selection process for the participants in this study.

The participants’ mean age was 67.7 ± 7.0 years. They were mostly female (84.9%), with an education level of more than four years of schooling (38.5%) and had low income (86.5% had a mean income of not more than four minimum monthly wages). Their mean pain intensity was 7.2 (± 2.6) on the numerical rating scale. The prevalence of severe low back pain-related disability was 54.5% (95% CI 50.5-58.4) for all participants, 54.6% (95% CI 50.2-58.9) for woman and 53.8% (95% CI 43.4-64.0) for men. The prevalence of disability was significantly higher among the elderly people with higher education level: 54.7% in the sample overall; 56.8% among the women, but 42.9% among the men. More details on the sociodemographic characteristics of the survey participants can be seen in Table 1.

**Table 1.** Sociodemographic and pain intensity characteristics of the sample of older adults complaining of acute low back pain, according to presence of disability, and with stratify according to sex. Back Complaints in the Elders - Brazil, 2016

Characteristic	Total sample (n = 602)	Disability^*^								
		All participants		P-value	Women	Severe (46.3%; n = 279)	P-value	Men	Severe (8.1%; n = 49)	P-value
		Not severe (45.5%; n = 274)	Severe (54.5%; n = 328)		Not severe (38.5%; n = 232)			Not severe (7.0%; n = 42)		
Age, median (SD)	67.7 (7.0)	68.1 (6.9)	67.3 (7.1)	0.157	67.9 (6.6)	67.5 (7.2)	0.483	69.4 (8.4)	66.4 (6.5)	0.060
Living, %										
With spouse/partner	44.9	43.6	46.0	0.548	35.6	39.8	0.466	82.9	81.6	0.873
Alone	55.1	56.4	54.0		63.4	60.2		17.1	18.4	
Education level, %										
< 4 years	38.5	30.3	45.3	< 0.001	30.4	43.2	0.003	29.3	57.1	0.008
4 or more years	61.5	69.7	54.7		69.6	56.8		70.7	42.9	
Income in minimum monthly wages, %										
Up to 1	39.7	34.7	44.0	0.070	35.1	45.3	0.061	32.5	36.2	0.806
2 to 4	46.8	50.9	43.3		52.0	42.8		45.0	46.8	
5 or more	13.5	14.4	12.7		13.0	12.0		22.5	17.0	
Pain intensity - NRS (0-10)	7.2 (2.6)	7.2 (2.5)	7.1 (2.7)	0.608	7.3 (2.6)	7.2 (2.7)	0.463	6.9 (2.3)	7.1 (2.6)	0.601

^*^Low back pain-related disability assessed using the Roland Morris Disability Questionnaire (RMDQ); SD = standard deviation; NRS = numerical rating scale.

Table 2 presents the characteristics of usage of healthcare services and therapeutic measures among all the participants and stratified according to sex and disability. Just 3.0% of these elderly people had consulted a physical therapist during the preceding six weeks, while 74.1% had used at least one analgesic. The results from bivariate analysis on the association between disability and indicators of use of healthcare services showed that the proportion of disability was more significant among those who had visited doctors during the preceding six weeks (35.4%), but not for men alone (30.6%). Among the indicators of therapeutic measures, the proportion of disability was higher among the elderly people who had used analgesics (78.1%) and was even higher among the women (79.9%).

**Table 2.** Bivariate analysis on the association between disability, indicators of use of healthcare services and indicators of therapeutic measures in the sample of older adults aged 60 years or over with complaints of acute low back pain. Back Complaints in the Elders - Brazil, 2016

Indicator	Total sample (n = 602)	Disability^*^								
		All participants^*^		P-value	Women		P-value	Men		P-value
		Not severe (n = 274)	Severe (n = 328)		Not severe (38.5%; n = 232)	Severe (46.3%; n = 279)		Not severe (7.0%; n = 42)	Severe (8.1%; n = 49)	
Use of healthcare services										
Doctor visits in the past 6 weeks^**^, %	31.2	26.3	35.4	0.017	25.9	35.2	0.012	28.6	30.6	0.832
Physiotherapy consultations in the past 6 weeks, %	3.0	2.6	3.4	0.567	2.6	3.2	0.670	2.4	4.1	0.658^a^
Diagnostic tests in the past 3 months, %	13.8	14.2	13.4	0.772	15.5	12.9	0.398	7.1	16.3	0.213^a^
Therapeutic measures										
Analgesic use, %	74.1	69.3	78.1	0.015	69.8	79.9	0.008	66.7	67.4	0.945
Complementary noninvasive therapy use^***^, %	13.6	13.1	14.0	0.752	11.6	15.4	0.217	21.4	6.12	0.059^a^

^*^Low back pain-related disability assessed using the Roland Morris Disability Questionnaire (RMDQ); ^**^Doctor visits included general practitioner, specialist and occupational doctors; ^***^Complementary noninvasive therapies included use of orthoses, braces, acupuncture, yoga, Pilates, overall postural re-education or other types; ^a^Fisher’s exact test.

Table 3 shows odds ratios for indicators of use of healthcare services and therapeutic measures due to low back pain-related disability for the total sample, adjusted for sociodemographic characteristic. The elderly people who visited doctors were 1.82 times (95% CI 1.22-2.71) more likely to present severe disability. The subjects who used analgesics were 1.57 times (95% CI 1.07-2.31) more likely to present disability. Women who visited doctors and used analgesics were significantly more likely to present severe disability. However, no significant association was found between the indicators of use of healthcare services and therapeutic measures and occurrences of severe disability in men. An additive effect was observed with regard to the chances of disability, in analyzing the combination of doctor visits and use of analgesics, both for the total sample (OR = 2.57; 95% CI 1.52-4.36) and for women (OR = 2.80; 95% CI 1.58-4.94).

**Table 3.** Multivariate logistic regression between indicators of use of healthcare services and therapeutic measures and occurrences of disability among older adults with complaints of acute low back pain. Back Complaints in the Elders - Brazil, 2016

Indicator	All participants^*^		Women^*^		Men^*^	
	OR (95% CI)	P-value	OR (95% CI)	P-value	OR (95% CI)	P-value
Doctor visits in the past 6 weeks^**^	1.82 (1.22-2.71)	0.003	2.00 (1.30-3.06)	0.002	0.77 (0.23-2.63)	0.681
Physiotherapy consultations in the past 6 weeks	1.07 (0.38-2.96)	0.902	0.97 (0.32-2.94)	0.963	2.82 (0.17-47.32)	0.472
Diagnostic tests in the past 3 months	0.72 (0.42-1.21)	0.217	0.60 (0.34-1.06)	0.076	3.53 (0.60-20.87)	0.165
Analgesic use	1.57 (1.07-2.31)	0.021	1.64 (1.08-2.50)	0.021	1.07 (0.36-3.16)	0.908
Complementary noninvasive therapy use^***^	1.27 (0.76-2.11)	0.361	1.60 (0.92-2.77)	0.094	0.26 (0.05-1.39)	0.115

OR = odds ratio; CI = confidence interval; ^*^Odds ratio and 95% confidence interval adjusted for age, living with spouse or partner, education level, income and pain intensity, as estimated through logistic regression; the exposure category was the low back pain-related disability and the response variables were the indicators of use of healthcare services and therapeutic measures; ^**^Doctor visits included general practitioner, specialist and occupational doctors; ^***^Complementary noninvasive therapies included use of orthoses, braces, acupuncture, yoga, Pilates, overall postural re-education or other types.

**Figure 2.**
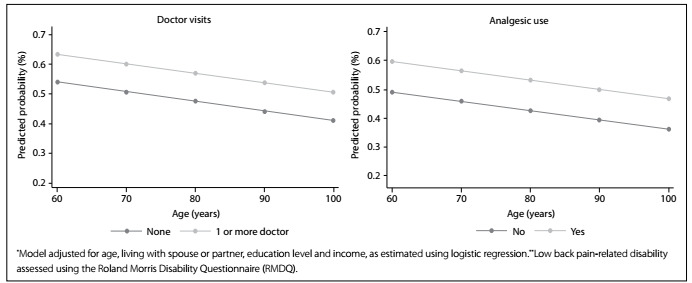
Predicted probability^*^ of visits to one or more doctor in the past six weeks and use of analgesics in the past three months, along the age continuum, according to disability^**^. Back Complaints in the Elders - Brazil, 2016.

Figure 2 shows that the predicted probability of doctor visits and use of analgesics was clearly stratified between different ages, with more significant probability among elderly people with severe disability.

## DISCUSSION

The results from this study showed that among elderly people, low back pain-related severe disability was associated with more significant chances of going to visit a doctor and using analgesics, even after taking into account potential confounding factors. Nevertheless, it should be noted this was more evident among women.

Doctor visits are a positive aspect of healthcare, since they provide opportunities for timely diagnosis, prevention, treatment and referral for rehabilitation.^19^ Perception of the severity of pain and, consequently, disability, may form the trigger for seeking medical attention.^28^^,^^29^ On the other hand, we expected that the elderly people who sought medical care and made use of analgesic medication would achieve relief from their pain symptoms and subsequently from the resulting disability. However, senescence and senility can cause higher demand for medical appointments, not only because of associations between pains and/or disability, but also because of involvement of the psycho-affective profile inherent to this age group. Among elderly people, stress, anxiety and depression are manifestations linked to occurrences of nonspecific low back pain and seeking medical appointments.^30^^,^^31^ Another reason for the more extensive use of healthcare services among elderly people with disabilities is their higher prevalence of chronic diseases and comorbidities, along with their complications, which lead to increased use of medications.^17^^,^^32^ Occurrences of psycho-affective factors and polypharmacy or other health conditions among elderly people can obscure accurate definition of the legitimate causal mechanisms that trigger severe symptoms of low back pain. This, which gives rise to a complex multidirectional relationship, means that it is sometimes impossible to distinguish temporal relationships from each manifestation, its diagnosis and the appropriate therapeutic approach.^33^ In addition, the overuse of healthcare services by elderly people has been shown to be a marker of care with only moderate resolution. Because of the complex nature of painful dysfunction, many elderly people are discharged from specialized rehabilitation services without any resolution for their pain symptoms and, thus, they continue to present disabilities.^34^

Medicines are widely prescribed at the beginning of the course of low back pain, although there is no consensus regarding recommendations for their use among elderly people.^11^^,^^35^^,^^36^ Our results have added to those in the literature, through understanding that inadequate management of healthcare can cause greater disabilities. The first line drugs recommended in the literature for reducing pain are analgesics and nonsteroidal anti-inflammatory drugs (NSAIDs).^37^^,^^38^ Nonetheless, even these drugs can result in side effects in elderly people. Their effectiveness remains unproven in relation to low back pain, compared with exercise, for example.^39^ Corroborating our results, increased risk of disability through chronic use of opioids within healthcare has been shown in the literature.^40^^,^^41^ Elderly people are more susceptible to the adverse effects of opioids, as has been shown through associations with falls and fractures,^42^ worse health outcomes, including worsening pain,^43^ increased likelihood of surgery and drug addiction after using painkillers for up to two years.^41^^,^^43^ Especially in older adults, important side effects associated with opioid use have been reported, including higher hospitalization risks, cardiovascular events, instability and cognitive impairment.^44^^,^^45^^,^^46^ Furthermore, common adverse effects such as bowel dysfunction, constipation, nausea and somnolence can put older adults at risk of injuries and losses of daily function.^47^

Our results showed that elderly users of analgesics were 1.57 times more likely to present low back pain-related disability. Furthermore, the combination analysis on medical consultations and analgesics demonstrated that these individuals presented 2.57 times higher chance of disability. Gold et al.^48^ demonstrated that elderly people with low back pain who received opioid medication early were 1.6 times more likely to have a new medical consultation and greater low back pain-related disability. Overuse of interventions and a “crowding-in effect”, defined as an additive combination of therapeutic and diagnostic measures,^49^ occur frequently among elderly people with low back pain.^29^ However, a more thorough understanding of perceived needs is necessary, to ensure effective results for patients and for the healthcare system. In a study on the use of healthcare services among 4,814 Italian elderly people, home and specialized care were used twice as often among those with disabilities.^49^ Chou et al.^14^ demonstrated that patients with nonspecific low back pain sought medical care in order to obtain a diagnosis and receive treatment options, but that they only showed concerns relating to pharmacotherapy. In the same study, the authors concluded that, so far, both non-pharmacological and pharmacological interventions have only been seen to have low to moderate effects in treatments for low back pain. Therefore, these issues need to be considered in developing approaches toward managing low back pain, to minimize negative effects like disability among elderly people.

Greater severity of disability was observed among women. In analyzing the associations with the interventions used, the results indicated that this stratification existed in relation both to doctor visits and to analgesic use. This hypothesis was confirmed in previous studies that demonstrated greater disability and worse prognosis for nonspecific low back pain among elderly women.^19^^,^^50^ The elderly population becomes preponderantly female, with consequently greater demand for healthcare services, and this has already been described in Brazil.^51^

The clinical course of differentiated low back pain among women reveals that they are at a disadvantage in relation to disability. Our results indicated that the frequency of low back pain-related disability among women was higher, but there was in addition a significant difference in the associations for doctor visits and analgesic use, in relation to disability. This may lead to the idea that the greater longevity, higher prevalence of low back pain and differences in musculoskeletal constitution and activities of daily living among women may constitute the key reasons that would explain the higher prevalence of disability and use of medical services in this subgroup.^1^^,^^6^^,^^52^

It is an established practice for individuals with low back pain to be referred to healthcare services such as diagnostic imaging, rehabilitation and other therapies, after the first visit to a doctor. A study by primary care physicians in Germany showed that the decision to refer a patient to specialized care was influenced by the characteristics of the healthcare system and the low back pain-related disability.^50^ In Brazil, the decision to refer patients for subsequent care apparently is made if treatment guidelines are not followed. Thus, no associations between disability and imaging diagnoses, consultations with physiotherapists or use of other noninvasive therapeutic measures were found in the present study. Likewise, Jarvik et al.^53^ showed that performing imaging examinations (radiography, magnetic resonance or computed tomography) did not produce differing results regarding low back pain-related disability among American elderly people. Therefore, the results from this study are consistent with the recommendation that performing these tests does not justify conclusions that lead to unnecessary interventions and harmful exposures.

Among the indicators of use of healthcare services, physical therapy consultations offer the potential to explain the incapacity. However, in the present investigation, as corroborated through the investigations of Loy et al.^54^ and Freburger et al.,^55^ there was no association between consultations with a physiotherapist and occurrences of low back pain-related disability. It is possible that this result was influenced by the low number of participants who had physical therapy consultation during the preceding six weeks (only 3%), which would demonstrate flaws in the medical referral system within public services and health plan services in Brazil and problems regarding timely access.^56^ Therefore, it can be concluded that the structure and funding of the healthcare system may have influenced the low demand for physical therapy assistance observed here. Medical-centered healthcare and a model based on overmedicalization could also explain the low frequency of use of non-invasive therapies as an alternative for treating low back pain and the associated disability.^13^^,^^14^^,^^17^^,^^56^ This information can be used by health promoters, policymakers and urban planners to support effective policies, programs and initiatives to promote effective access and therapeutic interventions.

### Limitations

The results from this study need to be considered cautiously. The cross-sectional design does not allow a temporal relationship to be established between the variables and the sample was predominantly composed of women, which may have led to underestimation of the results from the analyses between the subgroups. On the other hand, the present analysis was conducted on information that had been collected by trained professional interviewers using standardized instruments, which guaranteed the quality of the data. In addition, a careful analysis was carried out, considering potential confounding factors, according to the theoretical model. Thus, this study adds to the knowledge hitherto produced regarding the evidence from indicators of interventions associated with low back pain-related disability.

## CONCLUSIONS

In summary, the present study demonstrated that the combined effect of consultations and medication use was associated with higher chances of low back pain-related severe disability among elderly people. This suggested that overuse of medical services and “crowding-in” effects at these services were occurring among elderly people. This knowledge can contribute towards enabling more thorough understanding of occurrences of this disease. This favors planning of actions aimed at individual and collective monitoring of the population that is most vulnerable to this outcome, at least with regard to the indicators for the use of healthcare services and therapeutic measures in this population segment.
